# Acute Esophageal Necrosis: A Case Series From a Safety Net Hospital

**DOI:** 10.7759/cureus.8542

**Published:** 2020-06-10

**Authors:** Ishaan Vohra, Parth Desai, Kapil Thapa Chhetri, Hassam Shah, Anas Almoghrabi

**Affiliations:** 1 Internal Medicine, John H. Stroger, Jr. Hospital of Cook County, Chicago, USA; 2 Gastroenterology and Hepatology, John H. Stroger, Jr. Hospital of Cook County, Chicago, USA

**Keywords:** black esophagus, acute esophageal necrosis, endoscopy

## Abstract

Acute esophageal necrosis (AEN) or "black esophagus" is a rare clinical condition, endoscopically characterized by diffuse circumferential black esophageal mucosa. Etiologies include hypoperfusion, infections, and corrosive injury. Consideration of this condition as a differential diagnosis is important, as early diagnosis and treatment has implications on survival. In this article, we present four unique cases of acute esophageal necrosis who were managed conservatively.

## Introduction

Acute esophageal necrosis (AEN) is a rare, potentially lethal condition. Endoscopic findings show circumferential black esophageal discoloration in the distal one-third of the esophagus [1]. Distal esophageal involvement not extending beyond the gastroesophageal junction is the most common presentation. The esophagus lacks serosa which increases the risk of spread of the common infectious process to other organs. The entity follows a typical disease course divided into four separate stages (with stage 0 referring to a healthy esophagus) [2]. Stage 1 reflects acute inflammation with classic findings of the “black esophagus”. Stage 2 reflects a healing stage with friable mucosa covered with thick white-colored exudates and scant black areas. The esophagus renews its normal gross appearance in stage 3. Case reports have attributed the underlying etiology of AEN to widespread entities, including broad-spectrum antibiotics, diabetic ketoacidosis, underlying malignancy, lactic acidosis, peptic ulcer disease, malnourishment, infection, gastric outlet obstruction, prolonged vomiting, sepsis, ischemic processes, and trauma [3]. We describe four unique cases of AEN in an urban safety-net hospital.

## Case presentation

Case 1

A 72-year-old African-American male with a history of hypertension, diabetes mellitus, and stage IV pancreatic adenocarcinoma status post gastric-biliary bypass and chemoradiation therapy presented with a one-day history of coffee-ground emesis. On admission, he was afebrile and hemodynamically stable. Physical examination was remarkable for severe muscle wasting, cachexia, and abdominal distention with decreased bowel sounds. Laboratory examination revealed bicarbonate 19 mEq/L, chloride 98 mEq/L, lactate 3.8 mmol/L, anion gap 21, random blood glucose 212 mg/dL, blood urea nitrogen (BUN) 23 mg/dL, creatinine (Cr) 1.9 mg/dL, albumin 2.1 g/dL, white blood cell (WBC) 9.4 K/uL, hemoglobin 9.8 g/dL, and international normalized ratio (INR) 1.2. CT abdomen with oral contrast revealed gastric distention with a transition point at the level of duodenal bulb/pyloric region and large volume ascites.

**Figure 1:**
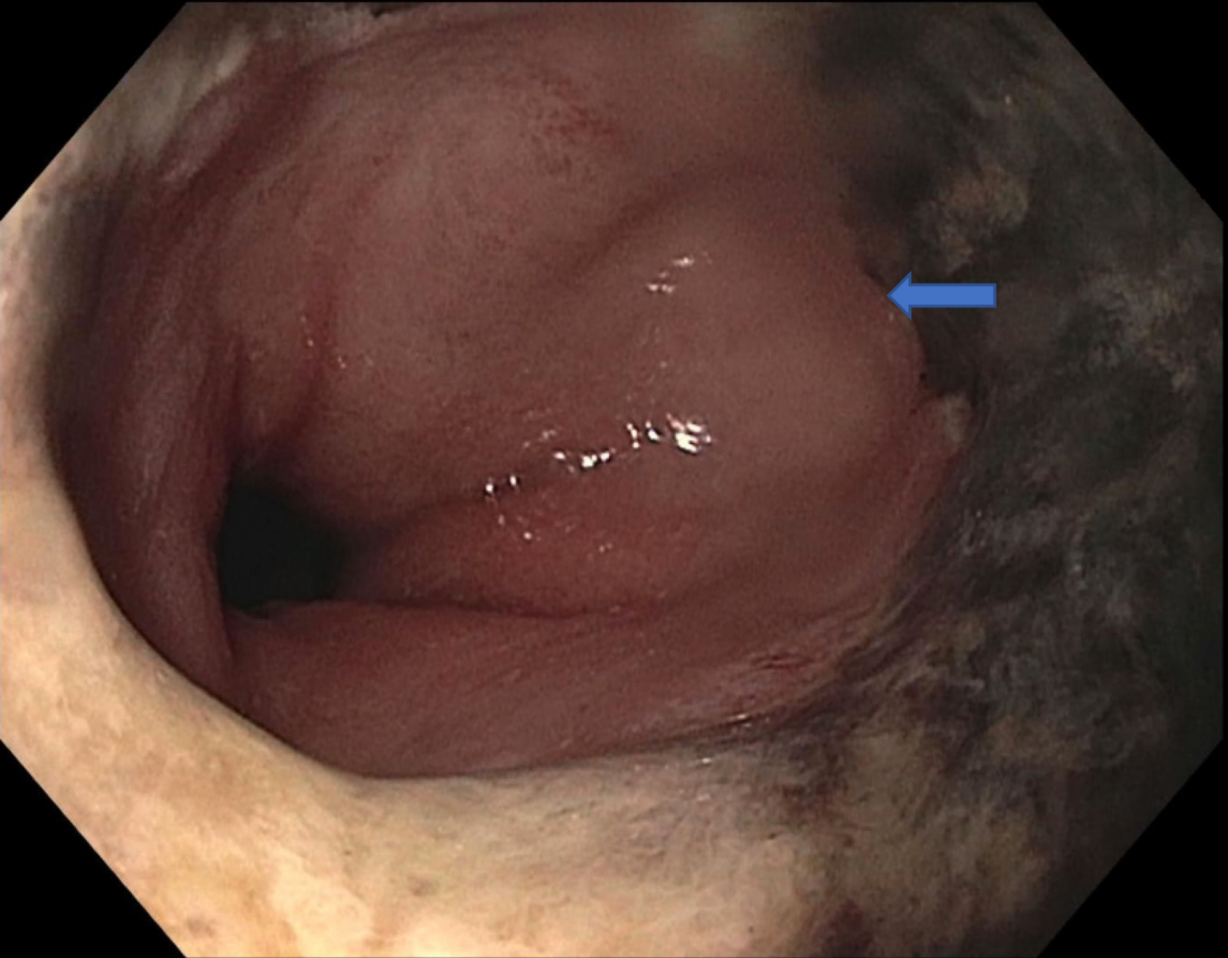
Circumferential black-colored discoloration suggestive of acute esophageal necrosis. Diffuse exudative esophagitis involving most of the esophagus extending from 23 cm to the gastroesophageal junction at 43 cm. Areas of black exudates were noted in the distal esophagus, with clear demarcation noted at the gastroesophageal junction (arrow).

He was initially suspected to have gastric outlet obstruction due to the imaging findings. Esophagogastroduodenoscopy (EGD) showed diffuse exudative esophagitis involving most of the esophagus with clear demarcation line between the necrotic esophagus and gastric mucosa (Figure 1).

**Figure 2:**
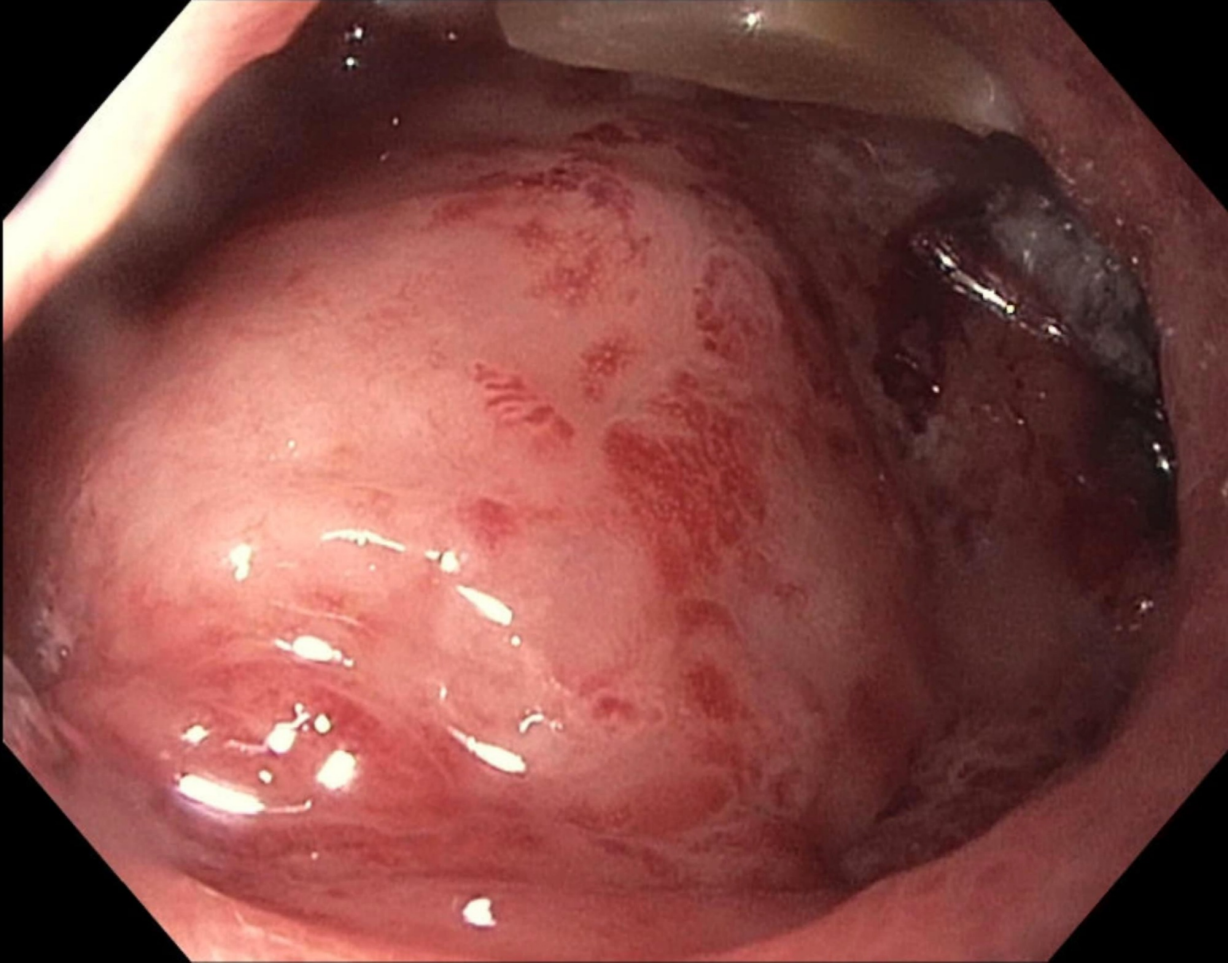
Pancreatic mass infiltrating and obstructing the duodenum. A large infiltrating mass, consistent with the known pancreatic cancer, was noted in the distal second part of the duodenum. It obstructed the duodenal lumen and endoscope could not be passed into the third part.

Biopsies were taken for pathology. Gastroenterostomy anastomosis from his prior surgery was noted in the distal stomach, which showed no luminal stenosis. A large infiltrating mass, consistent with the known pancreatic cancer, was noted in the distal second part of the duodenum, obstructing the duodenal lumen (Figure 2).

The patient was kept nil per os and was started on intravenous pantoprazole. Pathology report of esophageal biopsy showed fibrin-inflammatory debris. Special stain for Gomori methenamine silver (GMS) was positive for scattered budding yeasts, and rare fungal pseudohyphae or hyphae. The patient was prescribed a course of oral fluconazole to treat *Candida *esophagitis; however, he was lost to follow-up.

Case 2 

A 74-year-old male with no significant past medical history presented with coffee-ground emesis for five days as well as poor appetite and generalized weakness for three weeks. He denied using any non-steroidal anti-inflammatory drug use, tobacco, recent alcohol, or illicit drug usage. On admission, he was afebrile, tachycardic with a heart rate of 127 beats per minute, and hypotensive with a blood pressure 91/55 mmHg. On physical examination, he had severe muscle wasting. Laboratory examination revealed sodium of 129 mEq/L, potassium of 5.5 mEq/L, chloride of 93 mEq/L, bicarbonate of 11 mg/dL, anion gap of 25, lactate of 3.9 mmol/L. His BUN was 130 mg/dL and Cr 6.7 mg/dL. His hemoglobin was 12.4 g/dL with a WBC count of 20.5 K/uL with 96.4% neutrophils. Urinalysis showed large leukocyte esterase, six red blood cells (RBCs) per high-power field with five WBCs per high-power field.

**Figure 3:**
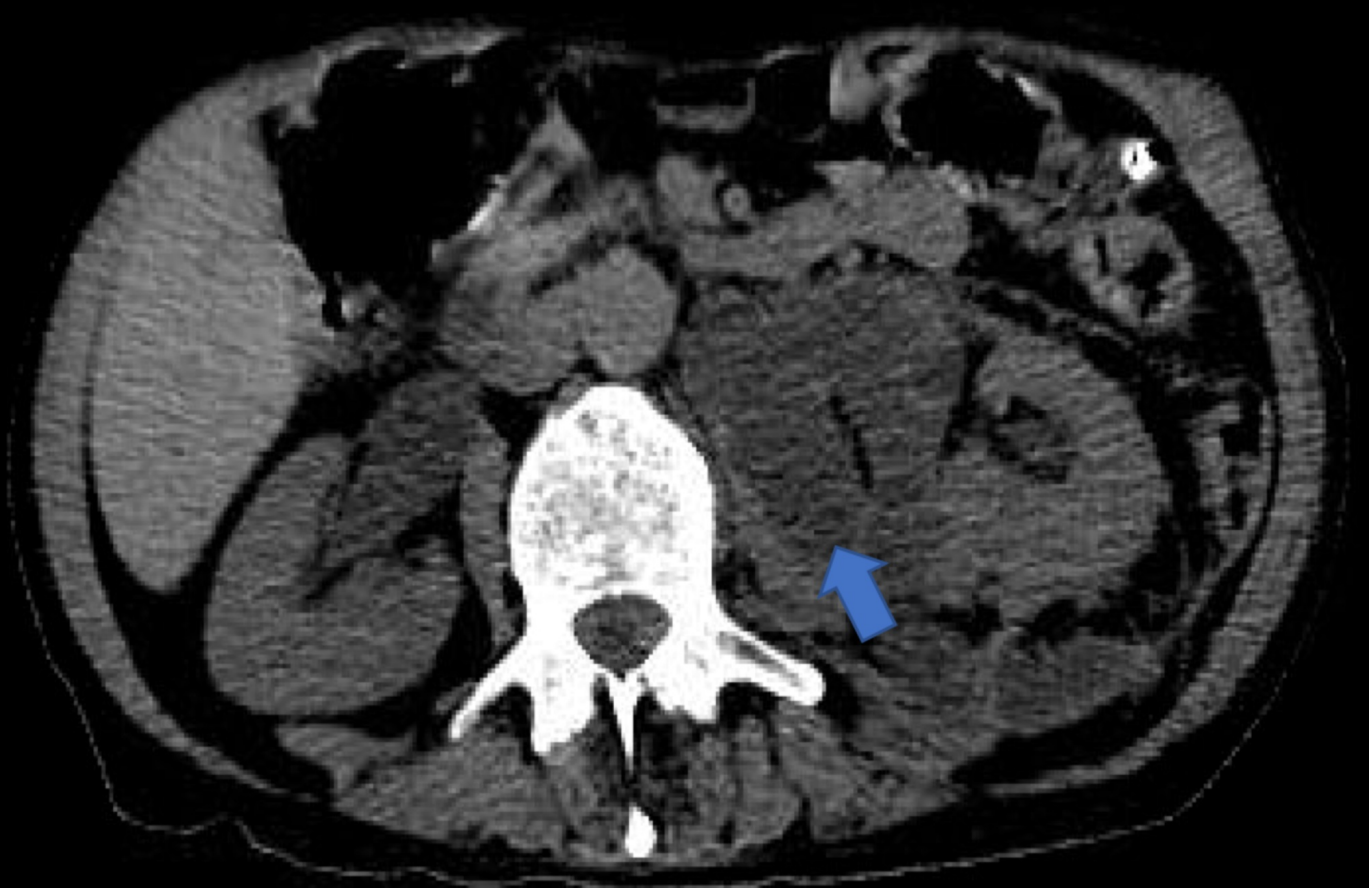
Multiloculated fluid in the posteromedial left perirenal space, extending to the renal sinus, as well as abscess along the left psoas muscle (blue arrow). Multiloculated fluid collection approximately measuring 8.0 x 3.0 x 10.4 cm extending medially to the renal sinus and encasing proximal ureter.

Non-contrast enhanced CT abdomen revealed multiloculated fluid in the posteromedial left perirenal space, extending to the renal sinus, as well as abscess along the left psoas muscle. He was transferred to the medical intensive care unit for upper gastointestinal bleeding and initially managed with intravenous fluids, intravenous pantoprazole, and octreotide drip. Additionally, he received intravenous antibiotics for intra-abdominal abscess (Figure 3, arrow)

A CT-guided drain catheter was placed for the loculated left perinephric fluid collection. EGD was performed which revealed severe diffuse Los Angeles (LA) grade D esophagitis in the entire esophagus and blackish discoloration of the distal esophagus. There was evidence of a Mallory-Weiss tear in the proximal stomach which was injected with epinephrine. Biopsies were not taken due to necrotic esophagus.

He improved with intravenous pantoprazole and fluids, and was kept nil per os. He was discharged home following cystoscopic left ureteral stent placement. Repeat EGD after 20 days revealed only a single distal esophageal ulcer at the gastroesophageal junction. Biopsies obtained revealed squamous epithelium with mild non-specific chronic inflammation and glandular mucosa (cardiac type) with acute and chronic inflammation. The cause of the AEN was thought to be due to severe esophagitis combined with a low flow state from hypotension on presentation.

Case 3 

**Figure 4:**
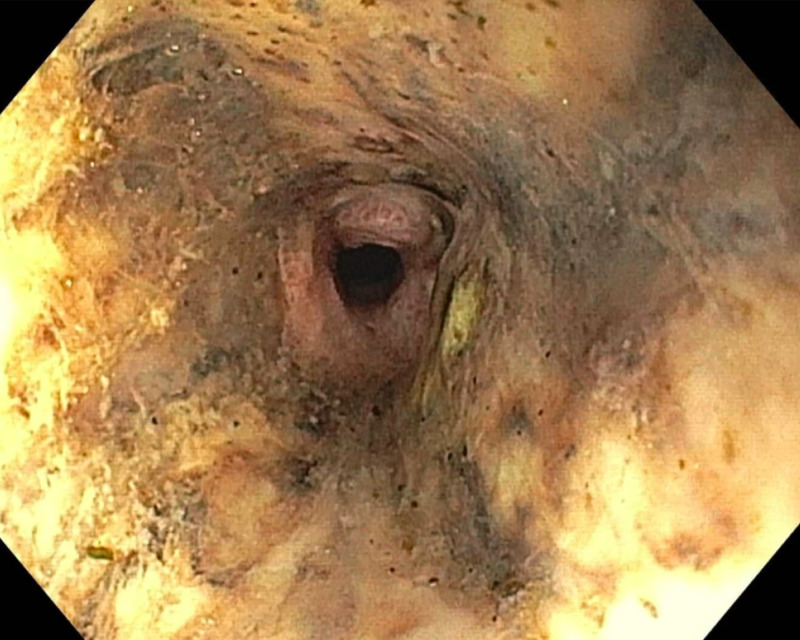
Esophagogastroduodenoscopy showing severe esophagitis in the entire esophagus and blackish discoloration of the distal esophagus. Starting at 25 cm from the incisors (mid esophagus) and extending all the way to the gastroesophageal junction (43 cm from the incisors), there was blackish discoloration of the esophagus consistent with acute esophageal necrosis.

A 62-year-old male with a history of multiple myeloma in remission complicated by end-stage renal disease and alcoholic liver cirrhosis presented to the emergency department with multiple episodes of non-bloody, non-bilious emesis for one week and dark stools. On admission, he was afebrile and hemodynamically stable, and abdominal exam revealed a non-distended, and non-tender abdomen. Laboratory examination revealed sodium of 149 mEq/L, potassium of 5.2 mEq/L, chloride of 71 mEq/l, bicarbonate of 49 mg/dL, anion gap of 29, and lactate of 1.8 mmol/L. His blood urea was 99 mg/dL and Cr 13.6 mg/dL. His hemoglobin was 15.3 g/dL and WBC count of 9,500 K/uL, which increased to 12.3 K/uL the following day. Coagulation profile was normal. He was found to have malfunctioning arteriovenous fistula, and symptoms were contributed to inadequate dialysis. He underwent fistulogram and angioplasty. Hemodialysis was performed, but his symptoms did not resolve. On the sixth day of admission, he had an episode of coffee-ground emesis with an acute drop in his hemoglobin of 3 g/dL. He was transferred to medical intensive care unit. He was managed medically for upper gastrointestinal bleeding. EGD was performed which revealed severe (LA grade D) esophagitis of the upper esophagus and blackish discoloration of the distal esophagus up to the gastroesophageal junction (Figure 4). 

Biopsies were not taken due to risk of perforating the necrotic, friable esophagus.

**Figure 5:**
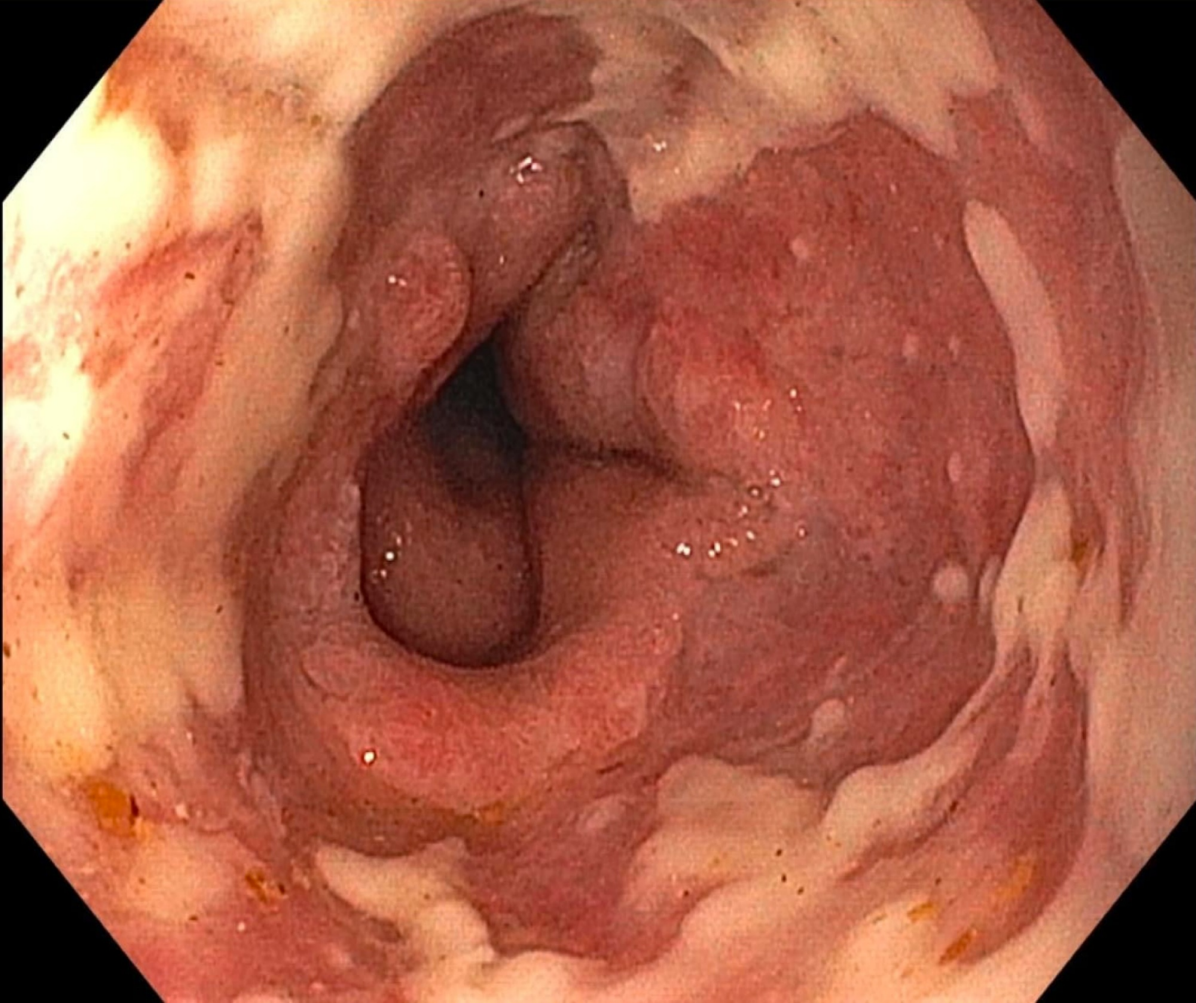
Circumferential esophagitis with exudates. Diffuse circumferential esophagitis with superficial ulceraion and exudates were noted throughout the esophagus to the gastroesophageal junction.

He was kept nil per os and managed with intravenous pantoprazole and fluids. His symptoms gradually improved. He also developed *Candida glabrata* fungemia and *Bacteroides thetaiotaomicron* due to possibly translocation from gastrointestinal tract, which was appropriately treated during his stay. Repeat EGD, performed 10 days later, showed diffuse circumferential esophagitis with superficial ulceration and exudates in distal esophagus (Figure 5).

**Figure 6:**
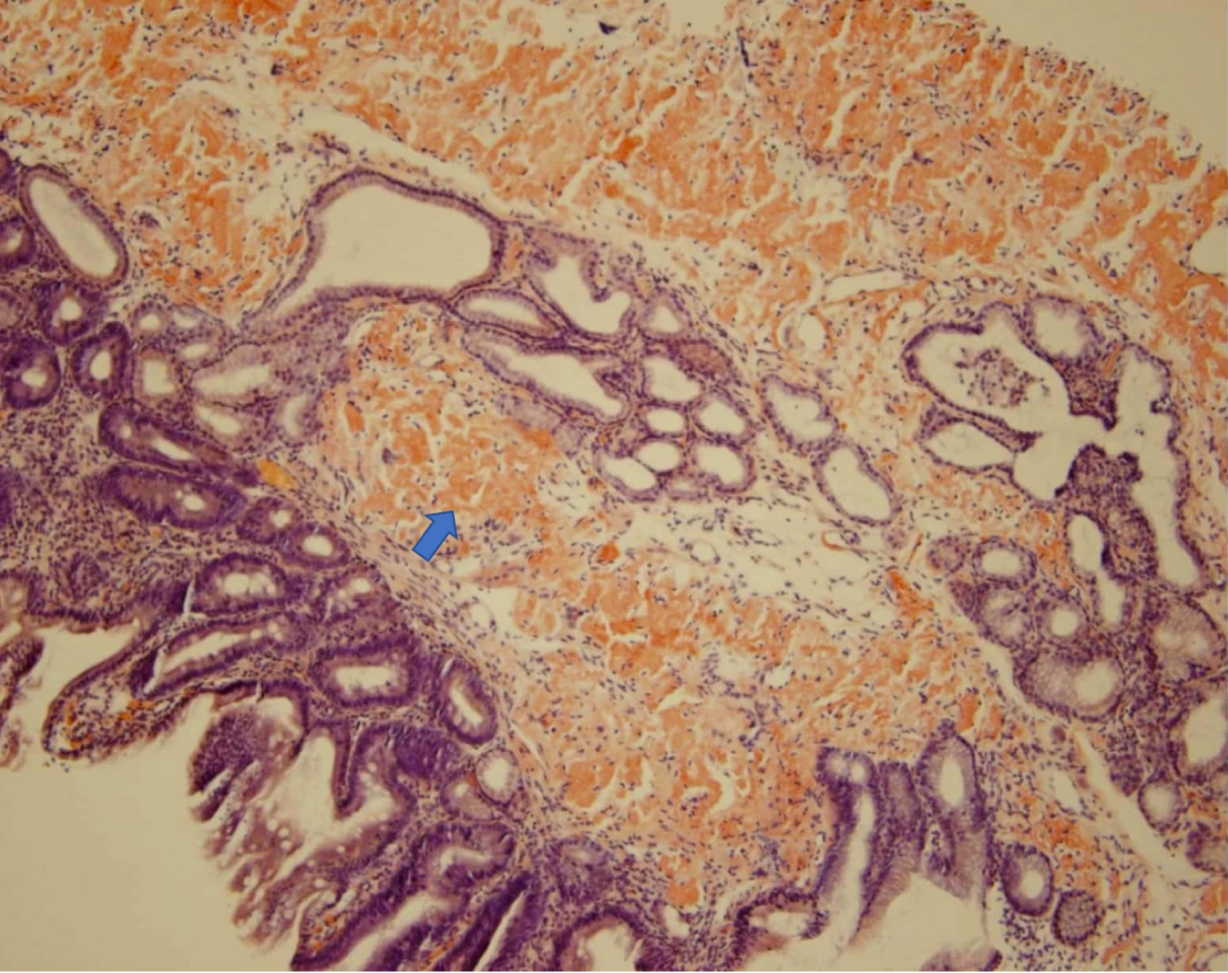
Duodenal biopsy showing amyloid deposition. Duodenal mucosa with amyloidosis and focal foreign body giant cell reaction (confirmed with Congo Red stain).

He had recurrent admissions for intractable nausea, vomiting, and poor oral intake over a span of two months. He was ultimately diagnosed with recurrence of myeloma and gastrointestinal amyloidosis based on repeat endoscopy-guided duodenal and colorectal biopsies (Figure 6).

The intractable vomiting and consequent acid exposure caused AEN in this patient. He was treated with bortezomib, dexamethasone, and cyclophosphamide for myeloma; however, he failed to respond and did not tolerate feeding trials. He ultimately chose to be transferred to hospice unit.

Case 4 

**Figure 7:**
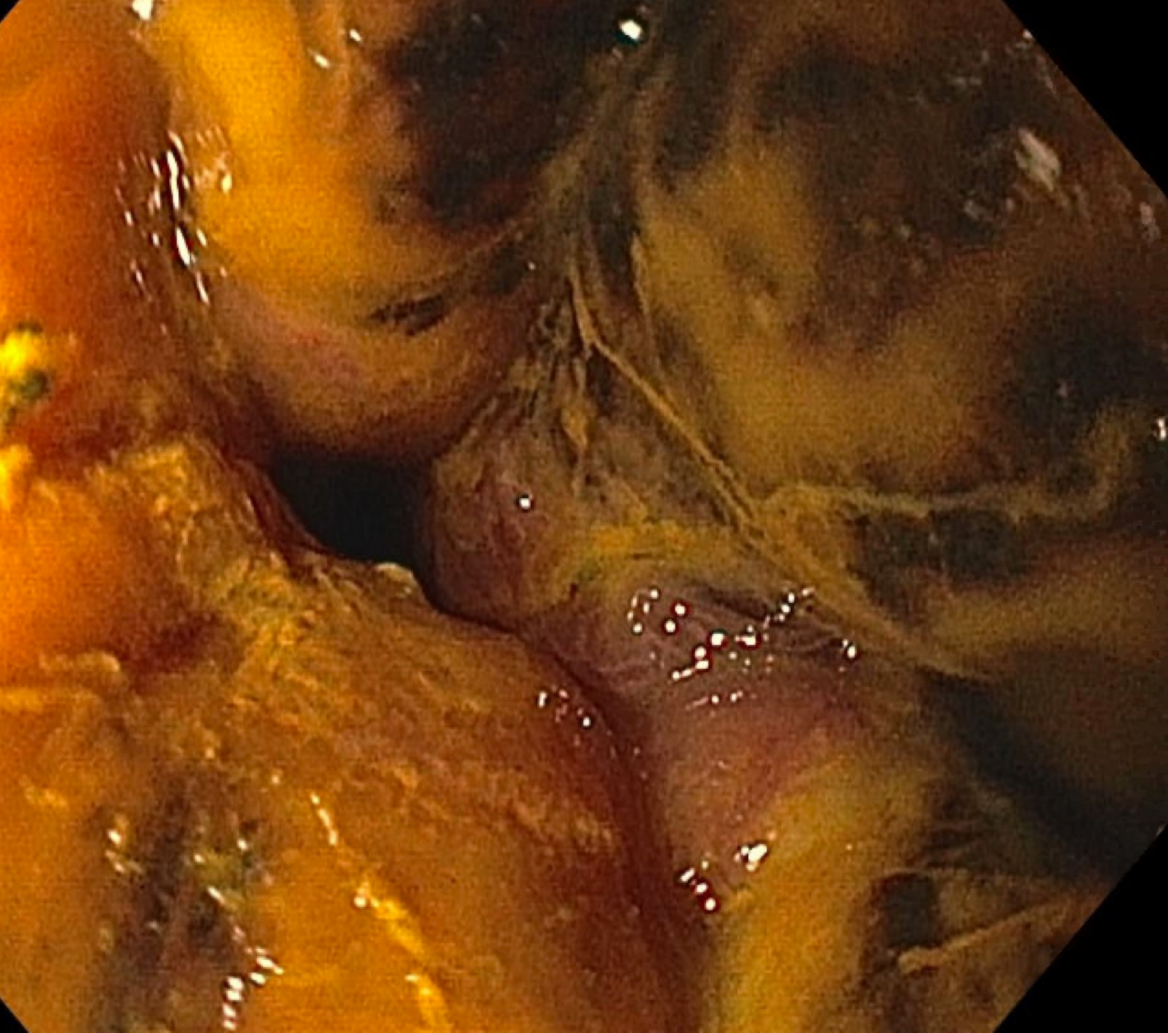
Esophagogastroduodenoscopy revealing esophageal necrosis. Diffuse esophagitis, with changes suggestive of black esophagus.

A 50-year-old Hispanic female with a history of hypertension presented with two days of abdominal pain, nausea, and multiple episodes of coffee-ground emesis. On admission, she was afebrile, tachycardic at 133 beats per minute, with an oxygen saturation of 90% on room air. On physical examination, she had dry oral mucosa, severe epigastric and right upper quadrant tenderness to palpation. Laboratory examination revealed serum creatinine of 2.3 mg/dL, BUN 44 mg/dL, WBC 16.1 K/uL, aspartate aminotransferase (AST) 118 U/L, and alanine aminotransferase (ALT) 279 U/L, with a serum lipase of 1,174 U/L. Contrast-enhanced CT abdomen revealed necrotizing pancreatitis with parenchymal necrosis, cholelithiasis with common bile ductal dilation up to 11 mm, and a non-compressive portal vein and splenic thrombosis. She was managed medically for necrotizing, gallstone pancreatitis and was kept nil per os. EGD was performed, revealing diffuse esophagitis up to the proximal/mid esophagus, with patches of blackish discoloration of the distal esophagus (Figure 7). 

Endoscopic retrograde cholangiopancreatography (ERCP) was attempted, but unsuccessful due to periampullary edema and ulceration. Her hospital course was complicated by hypoxic respiratory failure, altered mental status, and multiple right abdominal and peripancreatic fluid collections, requiring percutaneous drainage and perihepatic drain placement. Her peripancreatic fluid collection grew *Klebsiella oxytoca*, which was treated with intravenous ceftriaxone and metronidazole. Her symptoms improved, and she reported decreased abdominal pain and improved appetite. Her AEN was attributed to hypovolemia on presentation.

## Discussion

AEN is an endoscopic diagnosis, characterized by circumferential, black-appearing esophageal mucosa, usually involving the lower and mid esophagus, that stops abruptly at the gastroesophageal junction. Many studies have indicated that it may be relatively underdiagnosed. It is more common in elderly men, usually presenting in the sixth decade and is associated with multiple comorbid conditions. It is a rare clinical condition with a prevalence of 0.001% to 0.2%. The overall mortality of AEN is reported to be 34.5%, and most cases are fatal due to comorbidities, such as septic and cardiogenic shock [4].

AEN is a rare entity, esophageal biopsy or brushings of the affected esophageal tissue are supportive but not gold standard. Histological pathology shows evidence of absence of viable epithelium, abundant necrotic debris, and necrotic changes in the mucosa, possibly extending into the submucosa and even muscularis propria. Full-thickness necrosis has been described in the surgical specimen. Associated findings may include heavy leukocytic infiltrate, a combination of ischemic insult, corrosive injury from gastric content, and decreased function of the mucosal barrier systems. Reparative mechanisms have been proposed leading to AEN [2].

Most patients present with upper gastrointestinal bleeding, nausea, and vomiting. Odynophagia, dysphagia ,and epigastric pain can also be present. Physical examination may range from unremarkable vital signs to overt hemodynamic instability. The patient may appear cachectic, have pallor, fever, hypoxia, and abdominal tenderness, with guaiac-positive stools. Laboratory studies often show leukocytosis. Most findings are often due to the underlying comorbid condition and may include lactic acidosis, hypoalbuminemia, anemia, renal insufficiency, and hyperglycemia. Management involves treatment of the underlying comorbidity, acid suppression with intravenous proton pump inhibitor, typically includes nil per os for at least 24 hours, and intravenous hydration [2]. Sucralfate suspension has also been shown to be useful in preventing further injury [5]. Antimicrobial therapy plays an important role in case of positive esophageal cultures, stains for fungal agents, or visualization of multinucleated giant cells or inclusion bodies on histological evaluation of the biopsy specimen.

Complications of AEN include perforation, stenosis, and strictures [6]. Overall, the prognosis is poor, with a mortality rate of 13% to 35%, usually due to underlying comorbid conditions [7,8]. Surgery is indicated in patients with AEN with perforated esophagus, resulting in mediastinitis or abscess formation. Surgical options, such as esophagectomy, decortication, lavage, and delayed reconstruction, may be performed in addition to standard surgical approaches in the setting of gastric volvulus and transected thoracic aorta. Repeat endoscopy can be considered to assess for recovery and possible sequelae.

## Conclusions

AEN should be considered as a potential diagnosis of elderly men presenting with recurrent episodes of nausea, vomiting, and gastrointestinal bleeding. The development of necrotic esophagus remains a “red flag” and has high mortality due to underlying medical conditions. Therefore, AEN should be considered as a differential diagnosis warranting physicians to have knowledge and awareness on this serious clinical syndrome.
